# A simple rehabilitation regime improves functional outcome in patients with patellafemoral pain after 12 month

**DOI:** 10.1186/s40634-020-00223-z

**Published:** 2020-02-07

**Authors:** Torsten Grønbech Nielsen, Lene Lindberg Miller, Bjarne Mygind-Klavsen, Martin Lind

**Affiliations:** Orthopedic Department, Aarhus University Hospital, Palle Juul-Jensens Boulevard 99, 8200 Aarhus N, Denmark

**Keywords:** Patellofemoral pain, Knee pain, Patient education, Hip exercises

## Abstract

**Purpose:**

The purpose of the present study was to investigate the effects of a 3-month multimodal intervention including patient education, a simple hip exercise program, footwear adjustment, and foot orthoses to reduce symptoms in patients with patellafemoral pain (PFP).

**Methods:**

Patients were diagnosed based on a physical examination, patient symptoms and ruled out intra-articular knee pathologies by MRI. Patients were educated on PFP and participated in a 3-month exercise program; shoes with solid heel-caps were recommended, and custom made orthoses with arch support were recommended to patients with foot pronation. The Anterior Knee Pain Scale (AKPS) and the pain numeric rating scale (NRS) were used to evaluate the outcomes of the intervention and collected at baseline, 3 and 12-months follow-ups.

**Results:**

Sixty-five patients (age 18 years (9–32)) were included in a consecutive prospective cohort. The AKPS score improved from 71 ± 24 to 89 ± 9 (*p* < 0.01) at 12 months follow up. The NRS-rest and NRS-activity improved from 3 to 0 (p < 0.01) and 7 to 3 (p < 0.01) respectively. 78% of the patients clinically improved (i.e., demonstrated a > 10-point improvement (minimal clinically important difference (MCID))) considering the AKPS; and 76% and 73% clinically improved (i.e., demonstrated (MCID) a ≥ 2-point improvement) in their NRS-rest and NRS-activity, respectively. No patients experienced a decrease in their AKPS score or an increase in their NRS-rest and NRS-activity scores at 12-months.

**Conclusion:**

A 3-month PFP multimodal treatment strategy focusing on patient education, footwear adjustment, orthoses, and simple hip muscle exercises significantly improved functional outcomes and reduced pain at a 12 month follow-up.

## Background

Patellofemoral pain (PFP) is one of the of most common knee conditions in adolescents and young adults. The prevalence of knee pain in this age group varies from 9 to 40% [19]. The incidence of PFP varies from 4 to 7% and is approximately twice as high in females compared to males [3, 32].

Clinically, two types of patients with PFP are typically seen: the active patient who performed activities faster than their physiological capability and where the knee pain has lasted for a shorter period before consulting the clinic (< 12 months) and the sedentary patient who felt pain during activities of daily living (ADL) and where the duration of pain typically persisted for a longer period (> 12 months).

Many recent randomized controlled trials (RCTs) have compared different types of interventions, such as patella taping, orthoses, knee training, hip training, and education to evaluate which impact the interventions had to PFP patients [1, 4, 7, 10, 13, 15, 23, 24, 28].

The recommendations from The International Patellofemoral Research Network (iPFRN) in 2018 to reduce PFP include exercises (knee and hip) and a combined intervention including exercises and foot orthoses, patellar taping, or manual therapy [6].

Several studies with combined interventions have shown that patients experienced reduced pain from exercise therapy in the short-term, but this benefit was reduced in the long-term [2, 6, 13, 17, 21, 28]. Barton and colleagues recommended a multimodal treatment strategy including gluteal and quadriceps strengthening in combination with patella taping, activity modification, and patient education in the treatment of PFP patients [2]. Esculier et al. conducted a study including core and quadriceps training in combination with motor-control exercises and symptom management and patients experienced reduced pain and better knee function [12]. A RCT by Rathleff et al. compared exercise with patient education to patient education alone. They found that the combined intervention was superior [28].

The typical rehabilitation programs in studies demonstrating the effect of physiotherapy are protocols that include physiotherapist consultations 1–3 times per week for a period of 6–12 weeks, and each consultation lasts 30 min [5, 11, 13, 27, 28]. A simpler rehabilitation protocol could have an equal or better result due to better compliance, which has been shown to be a problem with intensive rehabilitation protocols [27].

This prospective cohort study included a simple hip exercise program combined with thorough patient education and footwear adjustment and was conducted to test the effectiveness of this treatment combination for patients with PFP.

The purpose of the present study was to investigate the effects of a minimal interfering 3-month multimodal intervention including patient education, a simple hip exercise program, footwear adjustment, and foot orthoses to reduce symptoms in patients with PFP. An age-related subgroup analysis illuminated whether there was a different response to the multimodal intervention in relation to the age of PFP patients.

It was hypothesized that PFP patients would benefit from a 3-month, simple, multimodal intervention at a 12-month follow-up. It was also hypothesized that there would be no age-related differences in the AKPS scores at the 12-month follow-up.

## Methods

### Patients selection

Patients were recruited from a single centre. Data was collected from October 2015 to April 2019. The Local Ethics Committee was contacted and determined that an approval for this study was not required (1–10–72-233-18).

Skilled orthopaedic surgeons diagnosed the patients based on a physical examination and patient symptoms and ruled out intra-articular knee pathologies as evaluated by MRI. Inclusion criteria: none-specific knee pain for minimum 3 months and no knee pathologies by MRi. Patients were seen by one of two physiotherapists at baseline, at 3 months, and at 12 months. Supplemental follow-up was provided after one month if the physiotherapist was unsure whether the patient could cope with the multimodal intervention at baseline.

### Treatment intervention

At baseline, all patients were instructed on the multimodal intervention, which included thorough patient education, three specific hip exercises, changes in footwear and the need for orthoses. The instruction and examination were conducted by a physiotherapist.

#### Patient education

- Patients were educated on correct foot-knee-hip alignment during activity of daily living (ADL) in relation to physical activities and training or exercises. The patients were instructed to focus on pointing the knee in the same direction as the pointer toe (second toe) whenever the foot was bearing weight.
- The patient typically felt pain during activities, such as cycling, running, climbing stairs, walking, and rising from a chair. Patients were educated on how to correctly perform those activities with neutral foot-knee-hip alignment.
- Controlled pain was only allowed during activities if the patient did not experience greater pain than was experienced before the activity. Pain during ADL should be kept at a minimum (i.e., an NRS-activity score of 3–4).
- Pain-inducing training (sport participation) was not recommended for a shorter period (mostly the first 3 month).

#### Hip exercises

**Fig. 1:**
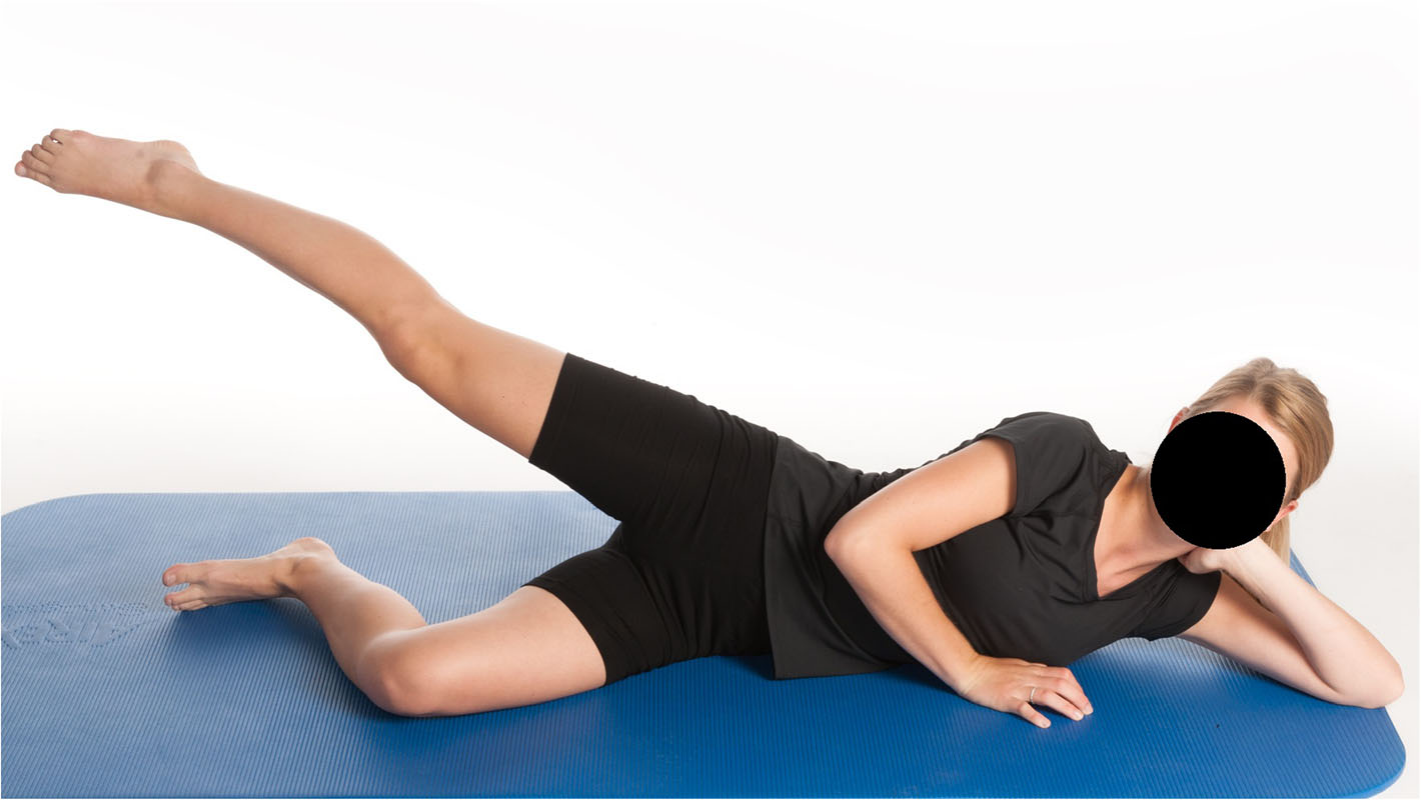
Straight leg side raises. The exercise should be performed on the floor with the pelvis tilted forward and the upper leg behind the plane of the body. Lift the straight upper leg and slowly returned it to the starting position

**Fig. 2:**
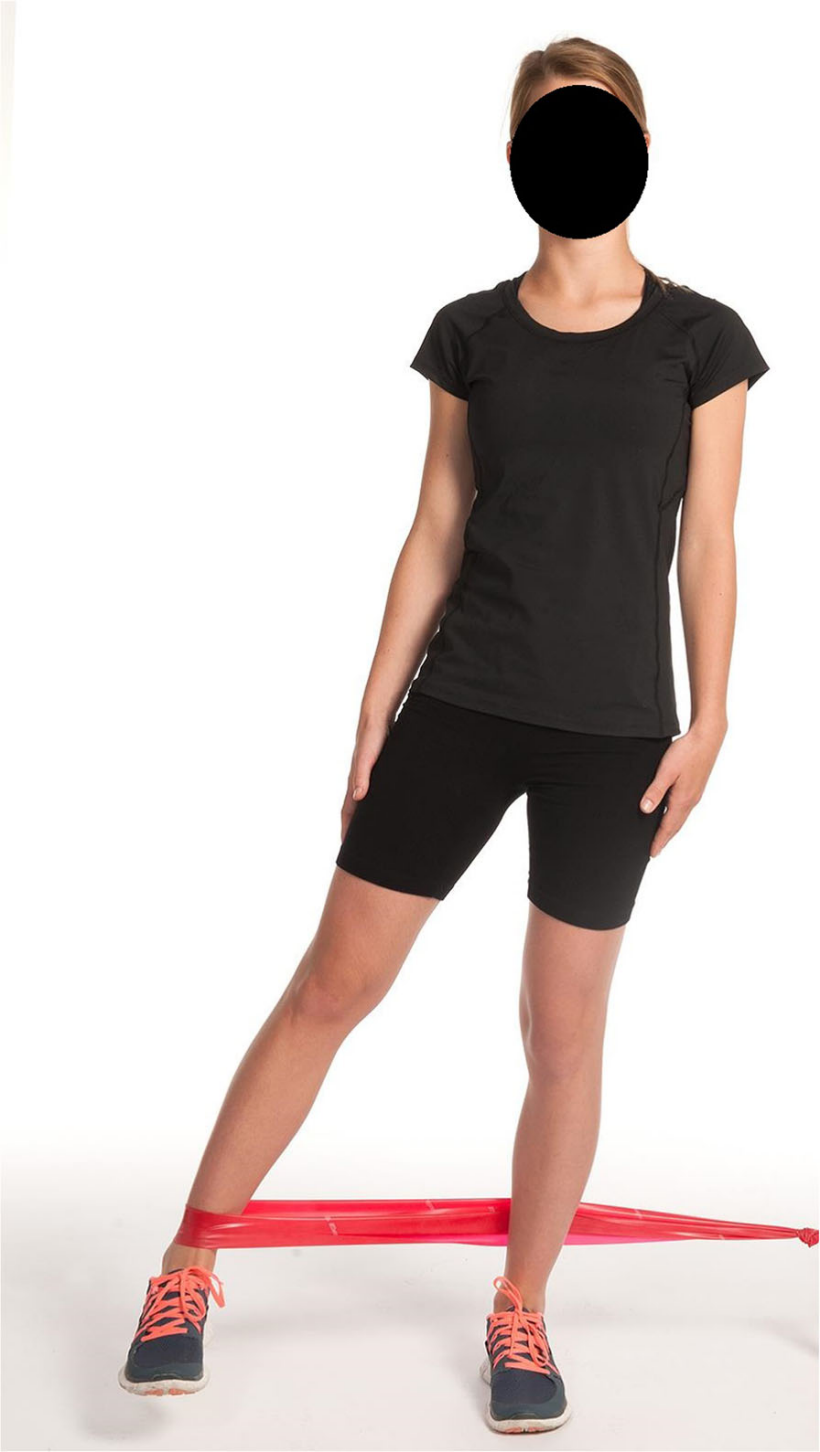
Standing hip abduction. In standing position with a resistance band wrapped around the ankle and fixed to a table or similar object.. Postural sways and the positive Trendelenburg position were not allowed during the exercise. Move the leg obliquely backwards and without pausing slowly returned it to the starting position. Should be done either barefoot or wearing shoes

**Fig. 3:**
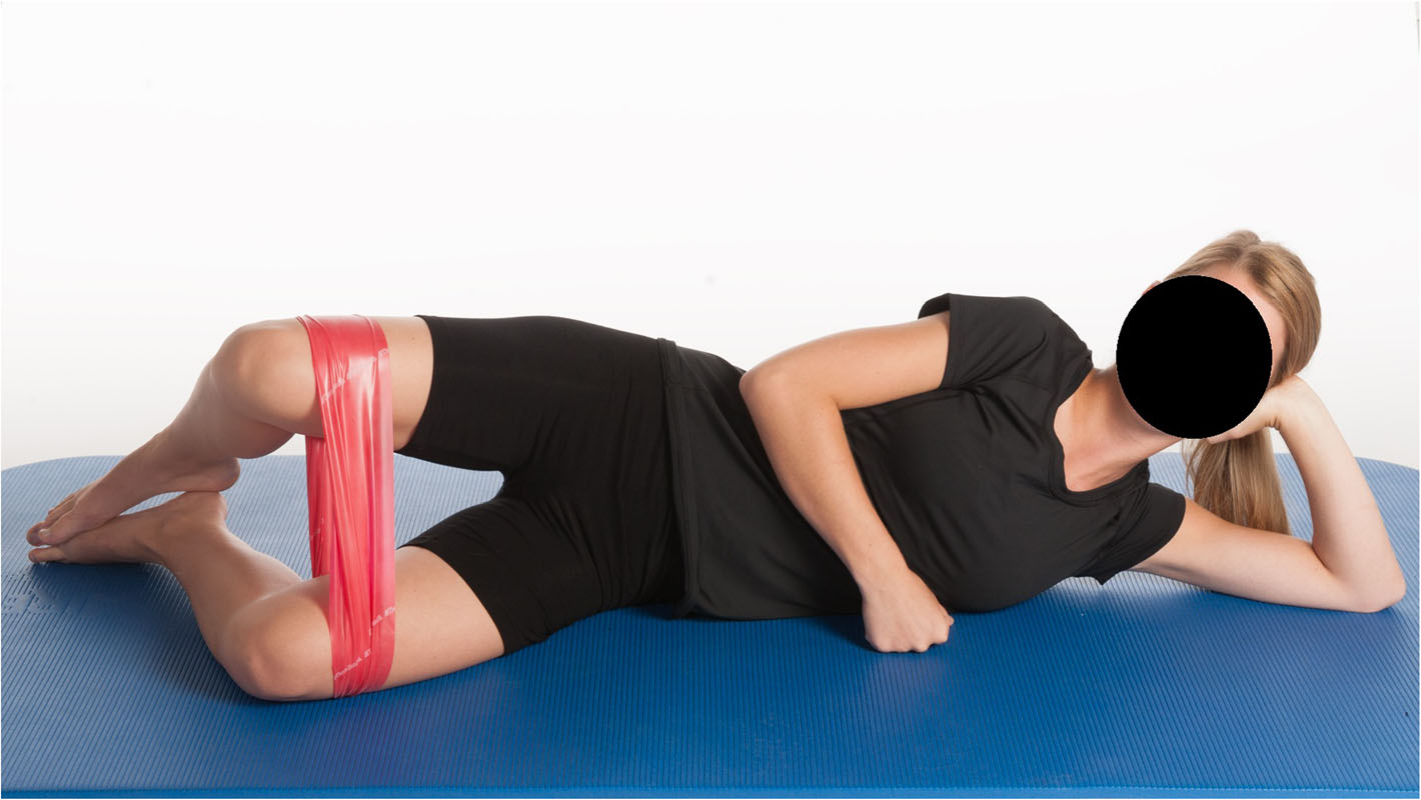
Clamshell exercise. Patient in a side lying position on the floor with their pelvis in a neutral position and the heels together. The upper knee was lifted as much as possible. A resistance band was placed proximal to the knee and allowed progression of the exercise

1. Straight leg side raises (hip abduction-gluteus medius) (Fig. 1)
  ○ The exercise was performed on the floor with the pelvis tilted forward and the upper leg behind the plane of the body.
  ○ The patient lifted the straight upper leg and slowly returned it to the starting position.
  ○ If the patient was not able to perform 10 repetitions, fewer repetitions were allowed. Correct form was essential.

2. Standing hip abduction (hip abduction-gluteus medius/core stability) (Fig. 2)
  ○ This exercise was done in a standing position with a resistance band wrapped around the ankle and fixed to a table or similar object. Resistance against hip abduction was sufficient so that the patient was able to perform 10–15 repetitions.
  ○ Postural sways and the positive Trendelenburg position were not allowed during the exercise.
  ○ The patient moved their leg obliquely backwards (45 degree angle between abduction and extension) and without pausing slowly returned it to the starting position.
  ○ The patient was allowed to either be barefoot or wear shoes depending on their preference.

3. Clamshell exercise (external hip rotation-gluteus medius/gemelli and obturator muscles) (Fig. 3)
  ○ The exercise was performed with the patients lying on their side on the floor with their pelvis in a neutral position and their heels together. One hand was placed on the pelvis to make sure that pelvis and trunk was not rotating during the exercise. Correct form was essential.
  ○ The upper knee was lifted as much as possible with the pelvis in a neutral position.
  ○ A resistance band was placed proximal to the knee and allowed progression of the exercise.

The exercises were performed daily (3 × 10–15 repetitions) and took approximately 10 min per day.

#### Footwear/orthoses (evaluated both with and without shoes)

1. To evaluate of the patients had pronated feet, patients were tested while standing and when doing single leg squats. If the midfoot arch collapsing during the test patients had pronated feet. We distinguish between pronated and normal feet. For the patients with pronated feet, orthoses were recommended to lift the pronated arch into a normal position. A prosthetist provided the patients with optimal orthoses. The orthoses were made of foam with an arch support and fit all shoes (EVA orthoses). The orthoses were all individually made.
2. Shoes with solid heel caps were recommended to patients with angle valgus and pronated feet. Screening for angle valgus was performed on barefoot patients in a standing position. Angle valgus was graded as pronated, neutral, or supine. If the ankle was pronated, shoes with solid heel caps were recommended.

A two-page leaflet with exercises and information on correct alignment were given to patients.

Patient education took approximately 15 min and included patients and relatives in a one-by-one session together with instructions for hip exercises and foot evaluation.

### Radiological evaluation

MRI was used to evaluate if the pain was caused by another intraarticular pathology, such as meniscus injury, cartilage injury, or osteochondritis dissecans, or other abnormal findings. To evaluate for dysplastic abnormalities, the tibial tuberosity-trochlear groove (TTTG), the sulcus angle, the Insall-Salvati ratio, and the Wiberg and Dejour dysplasia classification were applied [9, 16, 25, 31].

### Patient reported outcome scores (PROMS)

PFP was evaluated by the Anterior Knee Pain Scale (AKPS) [8, 20] and pain score (i.e., the Numeric Rating Scale at rest and during activity (NRS-rest and NRS-activity, respectively)).

The AKPS is a function score from 0 to 100; a high score indicates a good outcome. A 10-point increase was found to demonstrate a minimal clinically important difference (MCID) [8].

The NRS is a 10-point pain scale where 0 indicates no pain. A pain decrease of 2 points was found to demonstrate an MCID for the NRS pain scores [8].

Data were collected prior to the intervention and at the 3- and 12-month follow-ups by the physiotherapist.

### Statistical analyses

Normality was tested with QQ-plots. When data were normally distributed, the student T-test was used; otherwise the Mann-Whitney U test was performed to compare differences between groups. *P*-values below 0.05 were considered significant.

Two subgroup analyses were performed. First, patients were divided into three age groups: early adolescents (< 15 years), late adolescents (15–19 years), and adults (> 19 years). Second, patients were divided into activity groups related to their duration of pain before the intervention: the active group (< 12 months) and the sedentary group (> 12 months). Mann-Whitney U test was used for the subgroup analyses.

## Results

The present study was a prospective outcome study of 65 patients with a 12-month follow-up. The mean age was 18.1 years (range 9–32), and 80% of the patients were females. Patient characteristics are listed in Table 1.

**Table 1:** Patients demography

PFP Patients, n	65
Men/Women, %	20/80
Mean age in years (range)	18.1 (9–32)
Mean pain duration in months (range)	26 (3–72)
Data completeness	
• PROMS 3 Months	77%
• PROMS 12 Months	73%

There was no differences in demographic parameters between patients completed follow-up and patients who did not complete follow-up beside difference in mean age at 3 months follow-up. Patients who did complete the follow-up was 3.6 years older than the group of patients who did completed their 3 months follow-up (*p* = 0.02).

All patients had ankle valgus and were recommended to use shoes with a solid heel cap. Orthoses were recommended for 68% of the patients, but only 50% of these patients followed these recommendations.

MRI demonstrated no pathologies of menisci, ligaments or cartilage.

The AKPS scores significantly improved from 74 ± 14 at baseline to 84 ± 12 and 89 ± 9 at 3 and 12 months follow-up, respectively. The NRS scores significantly improved as well. 78% of the patients had a 10-point AKPS score improvement from baseline to 12 months (AKPS). Regarding NRS-rest and NRS-activity scores, a 2-point decrease in pain was seen in 76% and 73% of patients, respectively (Table 2).

**Table 2:** Outcome scores

	Baseline	3 months	*p-value* Baseline - 3 months	12 months	*p-value* Baseline − 12 months	*p-value* 3 months 12 months
AKPS	71 ± 14	84 ± 12*	< 0.01	89 ± 9*†	< 0.01	0.02
*- > 10-point improvement*		63%		78%		
NRS-rest (Ω)	3 (1–5)	0 (0–3)*	< 0.01	0 (0–1)*	< 0.01	0.33
*- > 2-point improvement*		68%		76%		
NRS-activity (Ω)	7 (5–8)	4 (2–6)*	< 0.01	3 (1–5)*	< 0.01	0.16
*- > 2-point improvement*		65%		73%		

Normally distributed data is presented as mean ± SD and non-normally data is presented as median (25th - 75th percentiles). Non-normally distributed data Ω

* Significant differences (*p* < 0.05) from baseline to follow-up at three months and from baseline to 12 months. † Significant difference from three to 12 months

The outcomes of differences related to age are shown in Table 3. At baseline, the adults’ AKPS scores were significantly better than the late adolescents’ scores. At 3 months, the adults had significantly less pain than the early adolescents according to the NRS-activity score. A success of a > 10-point improvement in AKPS from baseline to 12 months was seen in 71–81% of patients in all subgroups.

**Table 3:** Comparison of outcome scores in relation to age

	< 15 Years *(n = 16)*	15–19 years *(n = 29)*	> 19 years *(n = 20)*
AKPS			
*Baseline*	70 ± 18	69 ± 13	75 ± 10^a^
*3 months*	78 ± 17	84 ± 11^c^	87 ± 10 ^c^
*12 months*	90 ± 11^d^	89 ± 8^d^	89 ± 9^d^
*- > 10-point improvement*	80%	81%	71%
NRS-rest (Ω)			
*Baseline*	2 (1–5)	3 (1–5)	2 (2–5)
*3 months*	2 (0–4)	0 (0–3)^c^	0 (0–2)^c^
*12 months*	0 (0–1)	0 (0–2)^d^	0 (0–1)^d^
*- > 2 point improvement*	75%	62%	83%
NRS-Activity (Ω)			
*Baseline*	7 (5–9)	7 (6–9)	6 (4–7)
*3 months*	5 (4–7)	4 (3–6)^c^	3 (2–5)^b, c^
*12 months*	2 (0–5)^d^	4 (2–7)^d^	3 (1–5)^d^
*- > 2 point improvement*	83%	63%	67%
Data completeness			
*3 months, n(%)*	9 (56)	23 (79)	18 (90)
*12 months, n(%)*	12 (75)	20 (69)	16 (80)

Normally distributed data is presented as mean ± SD and non-normally data is presented as median (25th - 75th percentiles). Non-normally distributed data Ω

^a^significant difference between 15 and 19 years and > 19 years

^b^Significant difference between age < 15 and > 19 years

^c^Significant improvement from baseline to three months within subgroups

^d^significant improvement from baseline to 12 months within subgroups

No differences in the duration of pain were observed between the activity groups. The pain of patients in the active and the sedentary groups both significantly improved from baseline to the 3- and 12-month follow-up (Table 4).

**Table 4:** Comparison of outcome scores in relation pain duration

	< 12 Months *(n = 22)*	> 12 Months *(n = 36)*
AKPS		
*Baseline*	69 ± 12	71 ± 14
*3 months*	80 ± 15^a^	85 ± 10^a^
*12 months*	88 ± 10^b^	89 ± 8^b^
*- > 10-point improvement*	71%	81%
NRS-rest (Ω)		
*Baseline*	3 (1–4)	3 (1–5)
*3 months*	2 (0–4)	0 (0–2)^a^
*12 months*	0 (0–2)^b^	0 (0–1)^b^
*- > 2 point improvement*	73%	76%
NRS-Activity (Ω)		
*Baseline*	7 (5–8)	6 (5–8)
*3 months*	4 (3–6)^a^	3 (2–6)^a^
*12 months*	2 (0–5)^b^	3 (2–6)^b^
*- > 2 point improvement*	71%	73%

Normally distributed data is presented as mean ± SD and non-normally data is presented as median (25th - 75th percentiles). Non-normally distributed data Ω

^a^Significant improvement from baseline to 3 months within subgroups

^b^significant improvement from baseline to 12 months within subgroups

## Discussion

The primary finding of this study was that patients with PFP benefit from a simple multimodal intervention including patient education, a few daily exercises, and footwear adjustment.

An 18-point improvement in AKPS scores and a 3- to 4-point decrease in NRS scores from baseline to 12 months were equal to or greater than those seen in other studies with a more intensive training intervention [13, 14].

Ferber found a 12-point improvement after six weeks of either knee or hip/core training and a 2-point decrease in pain [13].

Fukado and colleagues found a 14-point improvement in the AKPS score and a 3-point decrease in pain at a 12-month follow-up in a knee-hip exercise study [14].

The studies mentioned above included three supervised physiotherapy sessions per week for six weeks (a total of 18 sessions). The patients in the study by Collins et al. attended six sessions over a 6-week period. Patients in the present study were seen once or twice during a 3-month period and performed exercises at home.

Training programs in the above-mentioned studies included more than eight exercises, including knee and hip strengthening and stretching. Patients in the present study were instructed to perform three strength exercises focusing on the hip. Those exercises were selected due to clinical experience and evidence-based literature [18, 22, 26, 30]. By choosing hip exercises, the focus on pain in the knee area was reduced.

Patient education is crucial to enable patients to learn about PFP and to empower them to resolve their knee problem. Patient education in the present study focused on movement optimization and using pain guidance for activity intensity. Patients were taught that pain helped them identify appropriate activities and exercises. Pain informs patients when to stop an activity and which activities are beneficial to avoid further pain. A principle of performing daily pain-free activities is the removal of the patients’ focus from their knee pain, and the avoidance of knee pain results in the patients experiencing fewer limitations in their activities.

Correction of footwear and/or orthoses might greatly impact patients with PFP and pronated feet and/or angle valgus. The recommendation from the newest consensus from iPFRN is that patients with PFP will benefit from orthoses in the short-term, and they should be used in combination with exercise therapy [6]. For this reason, the recommendation in this study was orthoses for patients with pronated feet.

The present study investigated the influence of age on the investigated treatment by dividing the patients into early adolescence, late adolescence, and adulthood groups. Only small differences were seen between groups, which showed that all age groups of patients with PFP will benefit from this multimodal treatment strategy. Rathleff et al. compared adolescents and adults and found that in the group of patients aged 15–19 years, only 1/3 of the patients had a successful outcome (7-point Likert scale) at a 12-month follow-up compared to 62–81% of the adults [29]. In the present study, 80% of the patients aged 15–19 years benefitted from this simple multimodal treatment.

Between the patients whose knee pain had persisted for a short period of time and those whose pain had persisted more than 12 months, this study showed no difference in outcome scores at baseline and the 3-month and 12-month follow-up. No other studies have performed this type of subgroup analysis, so these data are original in the discovery of a combined treatment being equally effective for patients with a short and long duration of symptoms. The 12-month cut-off was chosen due to the experience of the patient’s activity level before the intervention.

The present study did not clarify whether patients have the same benefits if one of the three interventions (i.e., exercise, education, and orthoses) was removed. Collins and colleagues showed that orthoses alone only reduce pain in the short-term [6]. Other studies found that combined interventions are better than only a single intervention [27, 28, 33]. Thus, a combined treatment strategy, as recommended by iPFRN, is important for optimal treatment outcomes in patients with PFP. The results suggest that a simpler and less resource exercise strategy has as good an outcome as more resource-extensive exercise programs.

We acknowledge several limitations of our study. The most important were the absence of a control group and the lack of a training diary. We cannot confirm if the patients performed the exercises as prescribed or if the patients followed the hip exercise program after 3 months. In addition, we do not know if patients used shoes with solid heel caps after the first 3 months or the duration they used the orthoses.

Another limitation of the study is the low follow up rate of 77% and 73% at 3 and 12 months follow-up, respectively.

Similarly, we do not know which of the three treatment modalities (i.e., exercise, footwear adjustment, or patient education) most significantly impacted the outcome.

## Conclusion

The present study demonstrates that PFP patients can be treated with a simple rehabilitation regimen focused on patient education, foot orthoses, and hip exercises. The exercises can primarily be performed outside physiotherapy clinic settings and with a clinical outcome comparable to other more resource-intensive exercise programs [29, 33]. This new and less resource-intensive rehabilitation program could be easier for PFP patients to include in their daily life compared to other regimens with similar documented clinical effects.

## Data Availability

The datasets used and/or analysed during the current study are available from the corresponding author on reasonable request.

## Abbrevations

ADL: Activities of daily living
AKPS: Anterior Knee Pain Scale
iPFRN: The International Patellofemoral Research Network
NRS: Pain numeric rating scale
PFP: Patellofemoral pain
RCTs: Randomized controlled trials
*TTTG*: *Tibial tuberosity-trochlear groove*
